# Crystal structure of 5,5′-bis­(di­methyl­amino)-*N*,*N*′-(3-methyl-3-aza­pentane-1,5-di­yl)di(naphthalene-1-sulfonamide)

**DOI:** 10.1107/S2056989015021714

**Published:** 2015-11-21

**Authors:** Toyketa V. Horne, Syed A. Haque, Adrianne Barton, Md. Alamgir Hossain

**Affiliations:** a Department of Chemistry and Biochemistry, Jackson State University, Jackson, MS 39217, USA

**Keywords:** crystal structure, sulfonamide, dansyl derivative, hydrogen bonding

## Abstract

In the title compound, C_29_H_37_N_5_O_4_S_2_, two arms substituted with dansyl derivatives are connected to a central tertiary amine, where the dihedral angle between the planes of two dansyl units is 56.39 (4)°. Each arm contains a sulfonamide functional group and both N—H groups in the compound are pointed to the same side. The central part of the mol­ecule is disordered over three sets of sites with a refined occupancy ratio of 0.547 (4):0.328 (4):0.125 (3). No intra­molecular π–π or hydrogen-bonding inter­actions are observed. In the crystal, mol­ecules are linked *via* pairs of N—H⋯O inter­actions involving the same acceptor atom, forming inversion dimers. In addition, C—H⋯O inter­actions exist between molecules, providing further stabilization of dimers.

## Related literature   

For general background to anion binding, see: Hossain (2008 ▸). For sulfonamide-based compounds as anion receptors, see: Kavallieratos *et al.* (2005 ▸). For related compounds, see: Basaran *et al.* (2015 ▸). For the anti­bacterial activity of sulfonamide-based compounds as drugs, see: Brackett *et al.* (2004 ▸).
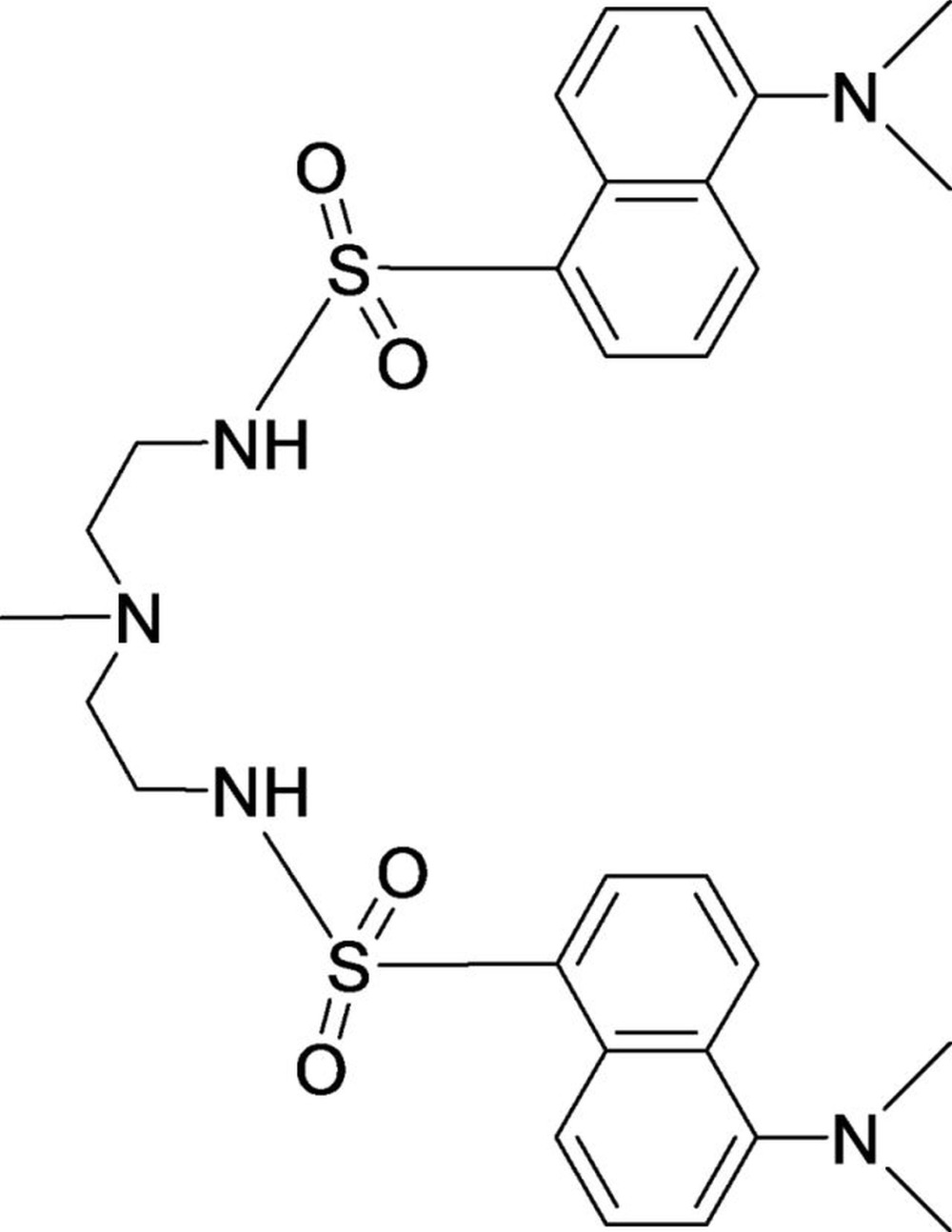


## Experimental   

### Crystal data  

C_29_H_37_N_5_O_4_S_2_
*M* *_r_* = 583.75
Triclinic, 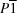

*a* = 10.5216 (5) Å
*b* = 11.3826 (5) Å
*c* = 13.9579 (6) Å
α = 107.8976 (7)°
β = 90.3662 (8)°
γ = 113.7328 (7)°
*V* = 1439.89 (11) Å^3^
*Z* = 2
Mo *K*α radiation
μ = 0.23 mm^−1^
*T* = 100 K
0.40 × 0.32 × 0.16 mm

### Data collection  

Bruker APEXII CCD diffractometer
Absorption correction: multi-scan (*SADABS*; Bruker, 2002 ▸) *T*_min_ = 0.914, *T*_max_ = 0.964
13576 measured reflections
7004 independent reflections
5916 reflections with *I* > 2σ(*I*)
*R*_int_ = 0.016

### Refinement  

*R*[*F* ^2^ > 2σ(*F* ^2^)] = 0.044
*wR*(*F* ^2^) = 0.122
*S* = 1.00
7004 reflections
466 parameters
655 restraints
H atoms treated by a mixture of independent and constrained refinement
Δρ_max_ = 1.06 e Å^−3^
Δρ_min_ = −0.71 e Å^−3^

### 

Data collection: *APEX2* (Bruker, 1998 ▸); cell refinement: *SAINT* (Bruker, 1998 ▸); data reduction: *SAINT*; program(s) used to solve structure: *SHELXT* (Sheldrick, 2015*a* ▸); program(s) used to refine structure: *SHELXL2014* (Sheldrick, 2015*b* ▸); molecular graphics: *XP* in *SHELXTL* (Sheldrick, 2008 ▸); software used to prepare material for publication: *SHELXL2014*.

## Supplementary Material

Crystal structure: contains datablock(s) global, I. DOI: 10.1107/S2056989015021714/ds2245sup1.cif

Structure factors: contains datablock(s) I. DOI: 10.1107/S2056989015021714/ds2245Isup2.hkl

Supporting information file. DOI: 10.1107/S2056989015021714/ds2245Isup3.cml

**Fig. 1.**
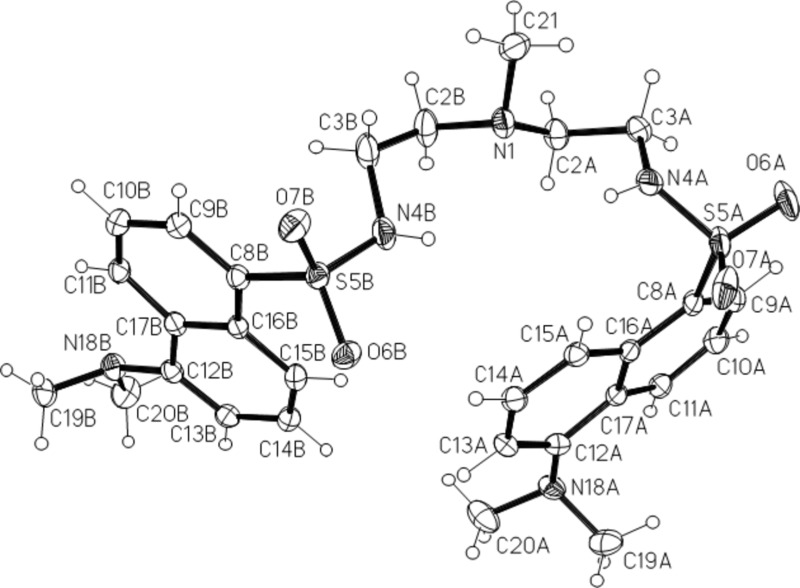
The molecular structure of the title compound (1) showing the atom-numbering scheme. Displacement ellipsoids are drawn at the 50% probability level (The minor components of the primed and double primed atoms are not shown for clarity).

**Fig. 2.**
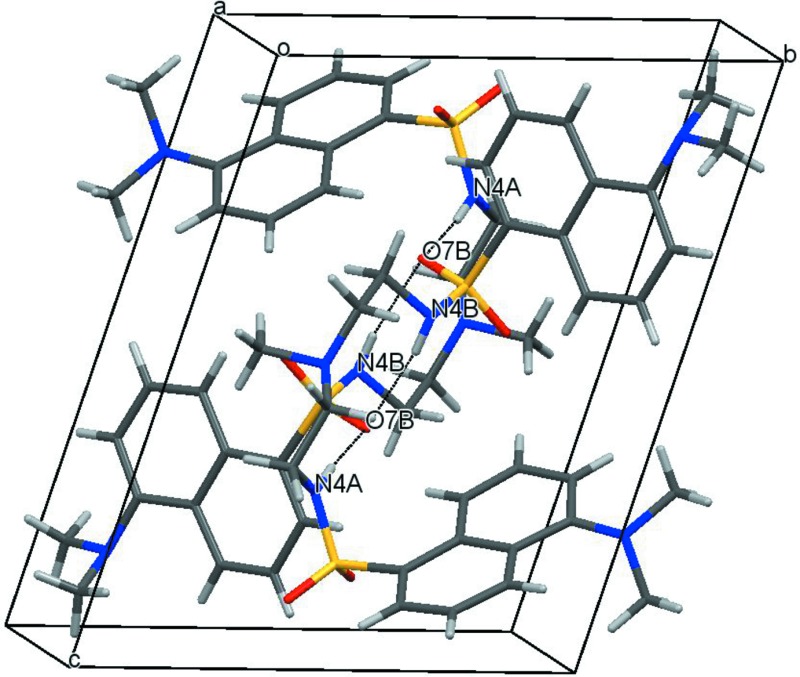
A unit cell of the title compound as viewed along the a axis showing hydrogen bonding interactions as dashed lines.

. DOI: 10.1107/S2056989015021714/ds2245fig1.tif
The mol­ecular structure of the title compound (1) showing the atom-numbering scheme. Displacement ellipsoids are drawn at the 50% probability level (The minor components of the primed and double primed atoms are not shown for clarity).

a . DOI: 10.1107/S2056989015021714/ds2245fig2.tif
A unit cell of the title compound as viewed along the *a* axis showing hydrogen bonding inter­actions as dashed lines.

CCDC reference: 1437206

Additional supporting information:  crystallographic information; 3D view; checkCIF report

## Figures and Tables

**Table 1:** Hydrogen-bond geometry (Å, °)

*D*—H⋯*A*	*D*—H	H⋯*A*	*D*⋯*A*	*D*—H⋯*A*
N4*A*—H4*A*⋯O7*B* ^i^	0.88 (2)	2.38 (2)	3.223 (2)	162 (2)
C20*A*—H20*B*⋯N4*A* ^ii^	0.98	2.60	3.494 (2)	152
C2*B*"—H2*B*6⋯O6*B* ^i^	0.99	2.54	3.359 (13)	140
N4*B*—H4*B*⋯O7*B* ^i^	0.84 (2)	2.41 (2)	3.2108 (19)	159 (2)
C9*B*—H9*B*⋯O7*A* ^i^	0.95	2.44	3.147 (2)	131
C21—H21*B*⋯O6*B* ^i^	0.98	2.65	3.203 (4)	116

Symmetry codes: (i) 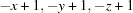; (ii) 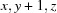.

## supplementary crystallographic information

### S1. Chemical context

Sulfonamide-based compounds are potential receptors for anions inter­acting *via* hydrogen bonding inter­actions under neutral conditions (Kavallieratos, *et al.* 2005). This class of compounds are also known to possess anti­bacterial activities and widely used as anti­biotics (Brackett *et al.* 2004). Previous studies suggest that tweezer-type compounds are effective in binding a variety of anions in solution (Basaran, *et al.* 2015). As a part of our ongoing research on anion chemistry (Hossain, 2008), we have been inter­ested in synthesizing sulfonamide-based neutral receptors. Herein, we report the crystal structure of the title compound, which is being studied in order to explore its ability to bind anions.

### S2. Structural commentary

The molecule adopts a bowl-like shape with the dihedral angle of 56.39 ° between the planes of two dansyl units. The hydrogen atoms of NH groups are positioned at the same side in the compound and are pointed inside the cavity formed by the NH—C—C—N—C—C—N chain of the central part (Fig. 1). TheC-N and C—C bond lengths in the aliphatic groups are in the range of 1-451 (3) to 1.491 (5) Å and 1.484(10 to 1.539 (4) Å, respectively; which are consistent with the literature value (Basaran, *et al.* 2015). The central part of the molecule containing N1, C2A, C3A, C2B, C3B, and C21 is disordered and chemically-equivalent distances are set to be approximately the same. Displacement parameters of disordered atoms are set to be approximately the same along each chemical bond. The sum of the three occupancies is set to 1.0. Details of the specific restraints are available in the *.cif. No intra­molecular π–π or hydrogen bonding inter­actions are observed. However, two molecules are linked *via* NH···O inter­actions [2.28 (2)–2.41 (2) Å] from two NH groups of one molecule with one oxygen group of other molecule (Fig. 2).

### S3. Synthesis and crystallization

2,2'-Di­amino-*N*-methyl­diethyl­amine (0.20 g, 1.71 mmol) and dansyl chloride (0.92 g, 3.43 mmol) were dissolved separately in 100 mL of CH_3_CN. The solution of dansyl chloride was added dropwise to the solution of 2,2'-di­amino-*N*-methyl­diethyl­amine containing K_2_CO_3_ (1.0 g) in a round bottom flask under constant stirring at room temperature. The mixture was allowed to stir over night at room temperature and the clear solution was separated by filtration. The solvent was evaporated under reduced pressure, and the product was purified by column chromatography on neutral alumina using 2% methanol in di­chloro­methane. The greenish yellow powder thus obtained was redissolved in methanol and crystals suitable for X-ray analysis were grown from the slow evaporation of the solvent.

### S4. Refinement

Crystal data, data collection and structure refinement details are summarized in Table 1. H atoms on C were placed in idealized positions with C—H distances 0.95 - 0.99 Å and thereafter treated as riding. The coordinates of those on N were refined. *U*_iso_ for H was assigned as 1.2 times *U*_eq_ of the attached atom (1.5 for methyl). A torsional parameter was refined for each methyl group. The largest residual density peak was 1.50 Å from O2.

### Crystal data

**Table:**

C_29_H_37_N_5_O_4_S_2_	*Z* = 2
*M**_r_* = 583.75	*F*(000) = 620
Triclinic, *P*1	*D*_x_ = 1.346 Mg m^−^^3^
*a* = 10.5216 (5) Å	Mo *K*α radiation, λ = 0.71073 Å
*b* = 11.3826 (5) Å	Cell parameters from 5579 reflections
*c* = 13.9579 (6) Å	θ = 2.3–28.3°
α = 107.8976 (7)°	µ = 0.23 mm^−^^1^
β = 90.3662 (8)°	*T* = 100 K
γ = 113.7328 (7)°	Block, yellow
*V* = 1439.89 (11) Å^3^	0.40 × 0.32 × 0.16 mm

### Data collection

**Table:**

Bruker APEXII CCD diffractometer	5916 reflections with *I* > 2σ(*I*)
φ and ω scans	*R*_int_ = 0.016
Absorption correction: multi-scan (*SADABS*; Bruker, 2002)	θ_max_ = 28.3°, θ_min_ = 1.6°
*T*_min_ = 0.914, *T*_max_ = 0.964	*h* = −14→14
13576 measured reflections	*k* = −14→15
7004 independent reflections	*l* = −17→18

### Refinement

**Table:**

Refinement on *F*^2^	Primary atom site location: structure-invariant direct methods
Least-squares matrix: full	Secondary atom site location: difference Fourier map
*R*[*F*^2^ > 2σ(*F*^2^)] = 0.044	Hydrogen site location: mixed
*wR*(*F*^2^) = 0.122	H atoms treated by a mixture of independent and constrained refinement
*S* = 1.00	*w* = 1/[σ^2^(*F*_o_^2^) + (0.066*P*)^2^ + 0.9*P*] where *P* = (*F*_o_^2^ + 2*F*_c_^2^)/3
7004 reflections	(Δ/σ)_max_ = 0.001
466 parameters	Δρ_max_ = 1.06 e Å^−^^3^
655 restraints	Δρ_min_ = −0.71 e Å^−^^3^

### Special details

**Table:**

Geometry. All e.s.d.'s (except the e.s.d. in the dihedral angle between two l.s. planes) are estimated using the full covariance matrix. The cell e.s.d.'s are taken into account individually in the estimation of e.s.d.'s in distances, angles and torsion angles; correlations between e.s.d.'s in cell parameters are only used when they are defined by crystal symmetry. An approximate (isotropic) treatment of cell e.s.d.'s is used for estimating e.s.d.'s involving l.s. planes.

### Fractional atomic coordinates and isotropic or equivalent isotropic displacement parameters (Å^2^)

**Table:**

	*x*	*y*	*z*	*U*_iso_*/*U*_eq_	Occ. (<1)
N1	0.1147 (4)	0.3189 (4)	0.5100 (2)	0.0241 (5)	0.547 (4)
C2A	0.0094 (3)	0.3376 (3)	0.5733 (2)	0.0253 (7)	0.547 (4)
H2A1	−0.0855	0.2755	0.5343	0.030*	0.547 (4)
H2A2	0.0198	0.4323	0.5897	0.030*	0.547 (4)
C3A	0.0225 (3)	0.3093 (4)	0.6725 (2)	0.0271 (9)	0.547 (4)
H3A1	−0.0416	0.3342	0.7175	0.032*	0.547 (4)
H3A2	−0.0017	0.2114	0.6580	0.032*	0.547 (4)
N1'	0.1135 (7)	0.2904 (5)	0.4924 (4)	0.0241 (5)	0.328 (4)
C2A'	0.0008 (5)	0.2427 (5)	0.5515 (3)	0.0274 (11)	0.328 (4)
H2A3	−0.0065	0.1562	0.5582	0.033*	0.328 (4)
H2A4	−0.0897	0.2246	0.5149	0.033*	0.328 (4)
C3A'	0.0285 (4)	0.3490 (6)	0.6581 (3)	0.0278 (11)	0.328 (4)
H3A3	0.0247	0.4312	0.6504	0.033*	0.328 (4)
H3A4	−0.0481	0.3114	0.6960	0.033*	0.328 (4)
N1"	0.1115 (12)	0.3352 (14)	0.5122 (7)	0.0241 (5)	0.125 (3)
C2A"	0.0560 (15)	0.2281 (10)	0.5579 (8)	0.0233 (17)	0.125 (3)
H2A5	0.1235	0.1877	0.5581	0.028*	0.125 (3)
H2A6	−0.0331	0.1551	0.5159	0.028*	0.125 (3)
C3A"	0.0297 (8)	0.2825 (13)	0.6672 (6)	0.0251 (17)	0.125 (3)
H3A5	−0.0416	0.3190	0.6688	0.030*	0.125 (3)
H3A6	−0.0026	0.2099	0.6981	0.030*	0.125 (3)
N4A	0.16734 (16)	0.39181 (14)	0.72099 (11)	0.0279 (3)	
H4A	0.236 (2)	0.419 (2)	0.6862 (17)	0.033*	
S5A	0.19585 (5)	0.48544 (4)	0.83930 (3)	0.02573 (11)	
O6A	0.10526 (17)	0.40288 (15)	0.89253 (11)	0.0402 (4)	
O7A	0.34484 (15)	0.54724 (15)	0.87101 (9)	0.0335 (3)	
C8A	0.14074 (17)	0.61568 (16)	0.84543 (11)	0.0199 (3)	
C9A	0.02852 (18)	0.61543 (17)	0.89534 (12)	0.0235 (3)	
H9A	−0.0156	0.5482	0.9261	0.028*	
C10A	−0.02093 (18)	0.71498 (17)	0.90082 (13)	0.0245 (3)	
H10A	−0.1004	0.7129	0.9334	0.029*	
C11A	0.04475 (17)	0.81468 (16)	0.85955 (12)	0.0215 (3)	
H11A	0.0103	0.8813	0.8642	0.026*	
C12A	0.23397 (16)	0.92570 (16)	0.76710 (12)	0.0205 (3)	
C13A	0.33375 (17)	0.91530 (16)	0.70590 (12)	0.0221 (3)	
H13A	0.3750	0.9806	0.6731	0.027*	
C14A	0.37514 (17)	0.80812 (16)	0.69159 (12)	0.0218 (3)	
H14A	0.4429	0.8017	0.6481	0.026*	
C15A	0.31982 (16)	0.71347 (16)	0.73883 (12)	0.0198 (3)	
H15A	0.3542	0.6463	0.7320	0.024*	
C16A	0.21055 (16)	0.71552 (15)	0.79828 (11)	0.0177 (3)	
C17A	0.16386 (16)	0.82035 (15)	0.80970 (11)	0.0184 (3)	
N18A	0.19170 (15)	1.03420 (14)	0.78728 (11)	0.0250 (3)	
C19A	0.2350 (2)	1.12772 (19)	0.89317 (15)	0.0341 (4)	
H19A	0.2088	1.0750	0.9394	0.051*	
H19B	0.1880	1.1887	0.9052	0.051*	
H19C	0.3369	1.1818	0.9055	0.051*	
C20A	0.2306 (2)	1.1122 (2)	0.71809 (17)	0.0378 (5)	
H20A	0.3328	1.1648	0.7295	0.057*	
H20B	0.1852	1.1745	0.7307	0.057*	
H20C	0.2001	1.0496	0.6476	0.057*	
C2B	0.0976 (7)	0.3461 (6)	0.4157 (3)	0.0358 (12)	0.547 (4)
H2B1	0.0632	0.4181	0.4297	0.043*	0.547 (4)
H2B2	0.0258	0.2624	0.3648	0.043*	0.547 (4)
C3B	0.2329 (14)	0.3911 (9)	0.3716 (4)	0.0338 (11)	0.547 (4)
H3B1	0.2751	0.3258	0.3647	0.041*	0.547 (4)
H3B2	0.2152	0.3962	0.3035	0.041*	0.547 (4)
C2B'	0.0841 (11)	0.3693 (8)	0.4368 (5)	0.0214 (12)	0.328 (4)
H2B3	0.0709	0.4456	0.4863	0.026*	0.328 (4)
H2B4	−0.0052	0.3100	0.3898	0.026*	0.328 (4)
C3B'	0.1951 (5)	0.4259 (6)	0.3774 (4)	0.0274 (13)	0.328 (4)
H3B3	0.2117	0.3507	0.3296	0.033*	0.328 (4)
H3B4	0.1624	0.4688	0.3368	0.033*	0.328 (4)
C2B"	0.1509 (13)	0.2805 (13)	0.4133 (8)	0.0245 (17)	0.125 (3)
H2B5	0.0647	0.2142	0.3645	0.029*	0.125 (3)
H2B6	0.2072	0.2313	0.4210	0.029*	0.125 (3)
C3B"	0.235 (6)	0.392 (3)	0.3706 (13)	0.030 (3)	0.125 (3)
H3B5	0.2937	0.3589	0.3244	0.036*	0.125 (3)
H3B6	0.1681	0.4038	0.3285	0.036*	0.125 (3)
N4B	0.32844 (15)	0.52834 (16)	0.44385 (11)	0.0282 (3)	
H4B	0.346 (2)	0.524 (2)	0.5015 (18)	0.034*	
S5B	0.47548 (4)	0.59859 (4)	0.40325 (3)	0.01928 (10)	
O6B	0.56111 (12)	0.72237 (12)	0.48413 (9)	0.0244 (2)	
O7B	0.53764 (14)	0.50402 (13)	0.36376 (9)	0.0277 (3)	
C8B	0.41900 (16)	0.63157 (16)	0.29830 (11)	0.0185 (3)	
C9B	0.41920 (17)	0.54760 (16)	0.20320 (12)	0.0209 (3)	
H9B	0.4509	0.4786	0.1966	0.025*	
C10B	0.37214 (17)	0.56457 (17)	0.11565 (12)	0.0229 (3)	
H10B	0.3696	0.5052	0.0500	0.027*	
C11B	0.33009 (17)	0.66641 (16)	0.12508 (12)	0.0215 (3)	
H11B	0.2968	0.6753	0.0655	0.026*	
C12B	0.29752 (16)	0.87008 (16)	0.23097 (12)	0.0195 (3)	
C13B	0.29406 (16)	0.95111 (16)	0.32655 (12)	0.0209 (3)	
H13B	0.2670	1.0231	0.3332	0.025*	
C14B	0.33008 (16)	0.92842 (16)	0.41442 (12)	0.0208 (3)	
H14B	0.3264	0.9854	0.4793	0.025*	
C15B	0.37033 (16)	0.82631 (16)	0.40870 (12)	0.0203 (3)	
H15B	0.3948	0.8136	0.4692	0.024*	
C16B	0.37556 (15)	0.73928 (15)	0.31206 (11)	0.0177 (3)	
C17B	0.33508 (15)	0.75903 (15)	0.22185 (12)	0.0182 (3)	
N18B	0.25959 (15)	0.88877 (15)	0.14093 (11)	0.0238 (3)	
C19B	0.37333 (19)	0.93504 (19)	0.08184 (13)	0.0277 (4)	
H19D	0.3333	0.9100	0.0110	0.042*	
H19E	0.4365	0.8916	0.0840	0.042*	
H19F	0.4260	1.0343	0.1111	0.042*	
C20B	0.1746 (2)	0.9649 (2)	0.15114 (15)	0.0327 (4)	
H20D	0.0969	0.9292	0.1877	0.049*	
H20E	0.1370	0.9554	0.0834	0.049*	
H20F	0.2328	1.0615	0.1892	0.049*	
C21	0.1058 (4)	0.1803 (3)	0.4852 (3)	0.0331 (8)	0.547 (4)
H21A	0.1307	0.1670	0.5476	0.050*	0.547 (4)
H21B	0.1709	0.1680	0.4374	0.050*	0.547 (4)
H21C	0.0098	0.1135	0.4541	0.050*	0.547 (4)
C21'	0.1317 (6)	0.1741 (5)	0.4216 (4)	0.0290 (12)	0.328 (4)
H21D	0.1432	0.1182	0.4592	0.044*	0.328 (4)
H21E	0.2152	0.2079	0.3895	0.044*	0.328 (4)
H21F	0.0489	0.1187	0.3691	0.044*	0.328 (4)
C21"	0.0074 (15)	0.3873 (14)	0.4998 (10)	0.032 (2)	0.125 (3)
H21G	0.0497	0.4645	0.4752	0.048*	0.125 (3)
H21H	−0.0231	0.4174	0.5655	0.048*	0.125 (3)
H21I	−0.0738	0.3148	0.4505	0.048*	0.125 (3)

### Atomic displacement parameters (Å^2^)

**Table:**

	*U*^11^	*U*^22^	*U*^33^	*U*^12^	*U*^13^	*U*^23^
N1	0.0199 (7)	0.0307 (12)	0.0206 (9)	0.0067 (8)	0.0042 (7)	0.0126 (9)
C2A	0.0192 (12)	0.0327 (15)	0.0254 (14)	0.0097 (11)	0.0057 (10)	0.0137 (11)
C3A	0.0285 (11)	0.0231 (11)	0.0252 (10)	0.0048 (7)	0.0106 (8)	0.0106 (8)
N1'	0.0199 (7)	0.0307 (12)	0.0206 (9)	0.0067 (8)	0.0042 (7)	0.0126 (9)
C2A'	0.0254 (19)	0.030 (2)	0.0216 (18)	0.0068 (17)	0.0072 (16)	0.0090 (16)
C3A'	0.0265 (13)	0.0255 (13)	0.0273 (13)	0.0080 (9)	0.0100 (9)	0.0073 (9)
N1"	0.0199 (7)	0.0307 (12)	0.0206 (9)	0.0067 (8)	0.0042 (7)	0.0126 (9)
C2A"	0.022 (3)	0.026 (3)	0.022 (2)	0.009 (2)	0.007 (2)	0.010 (2)
C3A"	0.0256 (19)	0.0254 (19)	0.0253 (18)	0.0113 (11)	0.0043 (10)	0.0092 (10)
N4A	0.0365 (8)	0.0201 (7)	0.0251 (7)	0.0098 (6)	0.0160 (6)	0.0079 (6)
S5A	0.0429 (3)	0.0278 (2)	0.0193 (2)	0.02282 (19)	0.01483 (17)	0.01416 (16)
O6A	0.0707 (10)	0.0405 (8)	0.0354 (7)	0.0363 (8)	0.0342 (7)	0.0292 (6)
O7A	0.0460 (8)	0.0525 (8)	0.0201 (6)	0.0365 (7)	0.0091 (5)	0.0149 (6)
C8A	0.0262 (8)	0.0204 (7)	0.0144 (7)	0.0114 (6)	0.0043 (6)	0.0055 (6)
C9A	0.0285 (8)	0.0248 (8)	0.0191 (7)	0.0117 (7)	0.0091 (6)	0.0093 (6)
C10A	0.0238 (8)	0.0285 (8)	0.0226 (8)	0.0127 (7)	0.0100 (6)	0.0082 (7)
C11A	0.0223 (7)	0.0226 (8)	0.0197 (7)	0.0111 (6)	0.0045 (6)	0.0052 (6)
C12A	0.0198 (7)	0.0177 (7)	0.0226 (7)	0.0077 (6)	0.0014 (6)	0.0055 (6)
C13A	0.0222 (7)	0.0187 (7)	0.0227 (8)	0.0048 (6)	0.0047 (6)	0.0087 (6)
C14A	0.0196 (7)	0.0206 (7)	0.0218 (8)	0.0065 (6)	0.0073 (6)	0.0054 (6)
C15A	0.0209 (7)	0.0181 (7)	0.0190 (7)	0.0087 (6)	0.0041 (6)	0.0040 (6)
C16A	0.0189 (7)	0.0186 (7)	0.0135 (6)	0.0074 (6)	0.0018 (5)	0.0036 (5)
C17A	0.0183 (7)	0.0187 (7)	0.0165 (7)	0.0073 (6)	0.0021 (5)	0.0045 (6)
N18A	0.0282 (7)	0.0180 (6)	0.0317 (8)	0.0119 (6)	0.0069 (6)	0.0093 (6)
C19A	0.0361 (10)	0.0236 (9)	0.0380 (10)	0.0145 (8)	0.0069 (8)	0.0021 (8)
C20A	0.0438 (11)	0.0292 (9)	0.0531 (12)	0.0199 (8)	0.0184 (9)	0.0246 (9)
C2B	0.0246 (18)	0.051 (2)	0.0222 (18)	0.0021 (16)	−0.0005 (16)	0.0185 (16)
C3B	0.0239 (17)	0.044 (2)	0.0211 (16)	−0.0004 (16)	0.0039 (14)	0.0149 (15)
C2B'	0.021 (2)	0.031 (2)	0.016 (2)	0.0130 (16)	0.0054 (19)	0.0095 (18)
C3B'	0.018 (2)	0.040 (3)	0.021 (2)	0.0059 (19)	0.0014 (18)	0.0146 (19)
C2B"	0.022 (3)	0.032 (3)	0.019 (2)	0.007 (2)	0.004 (2)	0.014 (2)
C3B"	0.023 (4)	0.041 (4)	0.020 (3)	0.003 (3)	0.004 (3)	0.015 (3)
N4B	0.0275 (7)	0.0354 (8)	0.0208 (7)	0.0069 (6)	0.0014 (6)	0.0171 (6)
S5B	0.02278 (19)	0.02060 (19)	0.01672 (18)	0.01048 (15)	0.00119 (14)	0.00761 (15)
O6B	0.0265 (6)	0.0243 (6)	0.0206 (6)	0.0111 (5)	−0.0027 (4)	0.0048 (5)
O7B	0.0406 (7)	0.0288 (6)	0.0213 (6)	0.0228 (6)	0.0005 (5)	0.0076 (5)
C8B	0.0193 (7)	0.0204 (7)	0.0169 (7)	0.0076 (6)	0.0016 (5)	0.0087 (6)
C9B	0.0232 (7)	0.0199 (7)	0.0199 (7)	0.0090 (6)	0.0040 (6)	0.0076 (6)
C10B	0.0270 (8)	0.0231 (8)	0.0170 (7)	0.0098 (6)	0.0031 (6)	0.0060 (6)
C11B	0.0237 (8)	0.0238 (8)	0.0167 (7)	0.0080 (6)	0.0013 (6)	0.0091 (6)
C12B	0.0171 (7)	0.0215 (7)	0.0203 (7)	0.0063 (6)	0.0016 (6)	0.0101 (6)
C13B	0.0198 (7)	0.0203 (7)	0.0238 (8)	0.0089 (6)	0.0037 (6)	0.0087 (6)
C14B	0.0205 (7)	0.0235 (8)	0.0179 (7)	0.0093 (6)	0.0038 (6)	0.0063 (6)
C15B	0.0208 (7)	0.0238 (8)	0.0178 (7)	0.0095 (6)	0.0037 (6)	0.0089 (6)
C16B	0.0164 (7)	0.0194 (7)	0.0171 (7)	0.0060 (6)	0.0026 (5)	0.0081 (6)
C17B	0.0166 (7)	0.0198 (7)	0.0177 (7)	0.0059 (6)	0.0025 (5)	0.0084 (6)
N18B	0.0272 (7)	0.0277 (7)	0.0222 (7)	0.0136 (6)	0.0020 (5)	0.0132 (6)
C19B	0.0339 (9)	0.0298 (9)	0.0209 (8)	0.0110 (7)	0.0021 (7)	0.0140 (7)
C20B	0.0400 (10)	0.0405 (10)	0.0295 (9)	0.0256 (9)	0.0009 (8)	0.0160 (8)
C21	0.0339 (17)	0.0251 (16)	0.0261 (18)	0.0036 (13)	0.0088 (14)	0.0021 (13)
C21'	0.026 (2)	0.030 (3)	0.032 (3)	0.015 (2)	0.005 (2)	0.008 (2)
C21"	0.032 (4)	0.036 (5)	0.034 (5)	0.017 (4)	0.008 (4)	0.015 (4)

### Geometric parameters (Å, º)

**Table:**

N1—C2A	1.463 (3)	C2B—C3B	1.512 (11)
N1—C2B	1.468 (3)	C2B—H2B1	0.9900
N1—C21	1.470 (4)	C2B—H2B2	0.9900
C2A—C3A	1.533 (4)	C3B—N4B	1.491 (3)
C2A—H2A1	0.9900	C3B—H3B1	0.9900
C2A—H2A2	0.9900	C3B—H3B2	0.9900
C3A—N4A	1.451 (3)	C2B'—C3B'	1.484 (10)
C3A—H3A1	0.9900	C2B'—H2B3	0.9900
C3A—H3A2	0.9900	C2B'—H2B4	0.9900
N1'—C2A'	1.466 (4)	C3B'—N4B	1.480 (3)
N1'—C2B'	1.467 (4)	C3B'—H3B3	0.9900
N1'—C21'	1.472 (5)	C3B'—H3B4	0.9900
C2A'—C3A'	1.539 (4)	C2B"—C3B"	1.52 (3)
C2A'—H2A3	0.9900	C2B"—H2B5	0.9900
C2A'—H2A4	0.9900	C2B"—H2B6	0.9900
C3A'—N4A	1.513 (3)	C3B"—N4B	1.484 (4)
C3A'—H3A3	0.9900	C3B"—H3B5	0.9900
C3A'—H3A4	0.9900	C3B"—H3B6	0.9900
N1"—C2A"	1.464 (4)	N4B—S5B	1.6273 (15)
N1"—C2B"	1.466 (4)	N4B—H4B	0.84 (2)
N1"—C21"	1.471 (6)	S5B—O6B	1.4350 (12)
C2A"—C3A"	1.533 (5)	S5B—O7B	1.4480 (12)
C2A"—H2A5	0.9900	S5B—C8B	1.7737 (15)
C2A"—H2A6	0.9900	C8B—C9B	1.377 (2)
C3A"—N4A	1.473 (4)	C8B—C16B	1.434 (2)
C3A"—H3A5	0.9900	C9B—C10B	1.411 (2)
C3A"—H3A6	0.9900	C9B—H9B	0.9500
N4A—S5A	1.6147 (15)	C10B—C11B	1.370 (2)
N4A—H4A	0.88 (2)	C10B—H10B	0.9500
S5A—O7A	1.4353 (15)	C11B—C17B	1.421 (2)
S5A—O6A	1.4355 (13)	C11B—H11B	0.9500
S5A—C8A	1.7766 (16)	C12B—C13B	1.380 (2)
C8A—C9A	1.374 (2)	C12B—N18B	1.418 (2)
C8A—C16A	1.435 (2)	C12B—C17B	1.440 (2)
C9A—C10A	1.408 (2)	C13B—C14B	1.408 (2)
C9A—H9A	0.9500	C13B—H13B	0.9500
C10A—C11A	1.369 (2)	C14B—C15B	1.370 (2)
C10A—H10A	0.9500	C14B—H14B	0.9500
C11A—C17A	1.426 (2)	C15B—C16B	1.424 (2)
C11A—H11A	0.9500	C15B—H15B	0.9500
C12A—C13A	1.377 (2)	C16B—C17B	1.436 (2)
C12A—N18A	1.426 (2)	N18B—C20B	1.456 (2)
C12A—C17A	1.438 (2)	N18B—C19B	1.470 (2)
C13A—C14A	1.413 (2)	C19B—H19D	0.9800
C13A—H13A	0.9500	C19B—H19E	0.9800
C14A—C15A	1.368 (2)	C19B—H19F	0.9800
C14A—H14A	0.9500	C20B—H20D	0.9800
C15A—C16A	1.426 (2)	C20B—H20E	0.9800
C15A—H15A	0.9500	C20B—H20F	0.9800
C16A—C17A	1.431 (2)	C21—H21A	0.9800
N18A—C20A	1.458 (2)	C21—H21B	0.9800
N18A—C19A	1.469 (2)	C21—H21C	0.9800
C19A—H19A	0.9800	C21'—H21D	0.9800
C19A—H19B	0.9800	C21'—H21E	0.9800
C19A—H19C	0.9800	C21'—H21F	0.9800
C20A—H20A	0.9800	C21"—H21G	0.9800
C20A—H20B	0.9800	C21"—H21H	0.9800
C20A—H20C	0.9800	C21"—H21I	0.9800
			
C2A—N1—C2B	110.5 (4)	H2B1—C2B—H2B2	107.8
C2A—N1—C21	111.4 (3)	N4B—C3B—C2B	106.5 (6)
C2B—N1—C21	109.6 (3)	N4B—C3B—H3B1	110.4
N1—C2A—C3A	112.3 (3)	C2B—C3B—H3B1	110.4
N1—C2A—H2A1	109.1	N4B—C3B—H3B2	110.4
C3A—C2A—H2A1	109.1	C2B—C3B—H3B2	110.4
N1—C2A—H2A2	109.1	H3B1—C3B—H3B2	108.6
C3A—C2A—H2A2	109.1	N1'—C2B'—C3B'	114.6 (6)
H2A1—C2A—H2A2	107.9	N1'—C2B'—H2B3	108.6
N4A—C3A—C2A	106.8 (2)	C3B'—C2B'—H2B3	108.6
N4A—C3A—H3A1	110.4	N1'—C2B'—H2B4	108.6
C2A—C3A—H3A1	110.4	C3B'—C2B'—H2B4	108.6
N4A—C3A—H3A2	110.4	H2B3—C2B'—H2B4	107.6
C2A—C3A—H3A2	110.4	N4B—C3B'—C2B'	112.0 (4)
H3A1—C3A—H3A2	108.6	N4B—C3B'—H3B3	109.2
C2A'—N1'—C2B'	110.7 (6)	C2B'—C3B'—H3B3	109.2
C2A'—N1'—C21'	110.4 (4)	N4B—C3B'—H3B4	109.2
C2B'—N1'—C21'	110.6 (4)	C2B'—C3B'—H3B4	109.2
N1'—C2A'—C3A'	111.2 (3)	H3B3—C3B'—H3B4	107.9
N1'—C2A'—H2A3	109.4	N1"—C2B"—C3B"	112.2 (15)
C3A'—C2A'—H2A3	109.4	N1"—C2B"—H2B5	109.2
N1'—C2A'—H2A4	109.4	C3B"—C2B"—H2B5	109.2
C3A'—C2A'—H2A4	109.4	N1"—C2B"—H2B6	109.2
H2A3—C2A'—H2A4	108.0	C3B"—C2B"—H2B6	109.2
N4A—C3A'—C2A'	115.1 (4)	H2B5—C2B"—H2B6	107.9
N4A—C3A'—H3A3	108.5	N4B—C3B"—C2B"	117.9 (15)
C2A'—C3A'—H3A3	108.5	N4B—C3B"—H3B5	107.8
N4A—C3A'—H3A4	108.5	C2B"—C3B"—H3B5	107.8
C2A'—C3A'—H3A4	108.5	N4B—C3B"—H3B6	107.8
H3A3—C3A'—H3A4	107.5	C2B"—C3B"—H3B6	107.8
C2A"—N1"—C2B"	109.2 (10)	H3B5—C3B"—H3B6	107.2
C2A"—N1"—C21"	110.9 (5)	C3B'—N4B—S5B	124.7 (3)
C2B"—N1"—C21"	110.3 (5)	C3B"—N4B—S5B	112.1 (13)
N1"—C2A"—C3A"	112.1 (5)	C3B—N4B—S5B	113.2 (4)
N1"—C2A"—H2A5	109.2	C3B'—N4B—H4B	119.1 (15)
C3A"—C2A"—H2A5	109.2	C3B"—N4B—H4B	112 (2)
N1"—C2A"—H2A6	109.2	C3B—N4B—H4B	111.0 (16)
C3A"—C2A"—H2A6	109.2	S5B—N4B—H4B	109.2 (15)
H2A5—C2A"—H2A6	107.9	O6B—S5B—O7B	116.27 (7)
N4A—C3A"—C2A"	104.2 (7)	O6B—S5B—N4B	106.76 (8)
N4A—C3A"—H3A5	110.9	O7B—S5B—N4B	111.90 (8)
C2A"—C3A"—H3A5	110.9	O6B—S5B—C8B	111.74 (7)
N4A—C3A"—H3A6	110.9	O7B—S5B—C8B	107.18 (7)
C2A"—C3A"—H3A6	110.9	N4B—S5B—C8B	102.12 (7)
H3A5—C3A"—H3A6	108.9	C9B—C8B—C16B	122.17 (14)
C3A—N4A—S5A	117.79 (16)	C9B—C8B—S5B	116.06 (12)
C3A"—N4A—S5A	124.1 (4)	C16B—C8B—S5B	121.77 (11)
C3A'—N4A—S5A	118.61 (19)	C8B—C9B—C10B	119.66 (15)
C3A—N4A—H4A	122.0 (15)	C8B—C9B—H9B	120.2
C3A"—N4A—H4A	119.6 (15)	C10B—C9B—H9B	120.2
C3A'—N4A—H4A	109.3 (15)	C11B—C10B—C9B	120.20 (15)
S5A—N4A—H4A	115.3 (15)	C11B—C10B—H10B	119.9
O7A—S5A—O6A	119.08 (9)	C9B—C10B—H10B	119.9
O7A—S5A—N4A	106.91 (8)	C10B—C11B—C17B	121.60 (14)
O6A—S5A—N4A	107.68 (9)	C10B—C11B—H11B	119.2
O7A—S5A—C8A	108.73 (8)	C17B—C11B—H11B	119.2
O6A—S5A—C8A	106.82 (8)	C13B—C12B—N18B	122.21 (14)
N4A—S5A—C8A	107.08 (8)	C13B—C12B—C17B	119.04 (14)
C9A—C8A—C16A	121.85 (15)	N18B—C12B—C17B	118.70 (14)
C9A—C8A—S5A	117.56 (12)	C12B—C13B—C14B	120.82 (15)
C16A—C8A—S5A	120.58 (12)	C12B—C13B—H13B	119.6
C8A—C9A—C10A	119.83 (15)	C14B—C13B—H13B	119.6
C8A—C9A—H9A	120.1	C15B—C14B—C13B	121.68 (15)
C10A—C9A—H9A	120.1	C15B—C14B—H14B	119.2
C11A—C10A—C9A	120.45 (15)	C13B—C14B—H14B	119.2
C11A—C10A—H10A	119.8	C14B—C15B—C16B	119.97 (14)
C9A—C10A—H10A	119.8	C14B—C15B—H15B	120.0
C10A—C11A—C17A	121.23 (15)	C16B—C15B—H15B	120.0
C10A—C11A—H11A	119.4	C15B—C16B—C8B	124.11 (14)
C17A—C11A—H11A	119.4	C15B—C16B—C17B	118.78 (14)
C13A—C12A—N18A	122.92 (15)	C8B—C16B—C17B	117.10 (13)
C13A—C12A—C17A	119.35 (14)	C11B—C17B—C16B	119.09 (14)
N18A—C12A—C17A	117.68 (14)	C11B—C17B—C12B	121.28 (14)
C12A—C13A—C14A	120.41 (14)	C16B—C17B—C12B	119.62 (14)
C12A—C13A—H13A	119.8	C12B—N18B—C20B	115.74 (14)
C14A—C13A—H13A	119.8	C12B—N18B—C19B	116.12 (13)
C15A—C14A—C13A	121.53 (14)	C20B—N18B—C19B	111.02 (14)
C15A—C14A—H14A	119.2	N18B—C19B—H19D	109.5
C13A—C14A—H14A	119.2	N18B—C19B—H19E	109.5
C14A—C15A—C16A	120.03 (14)	H19D—C19B—H19E	109.5
C14A—C15A—H15A	120.0	N18B—C19B—H19F	109.5
C16A—C15A—H15A	120.0	H19D—C19B—H19F	109.5
C15A—C16A—C17A	118.71 (14)	H19E—C19B—H19F	109.5
C15A—C16A—C8A	123.84 (14)	N18B—C20B—H20D	109.5
C17A—C16A—C8A	117.42 (13)	N18B—C20B—H20E	109.5
C11A—C17A—C16A	119.04 (14)	H20D—C20B—H20E	109.5
C11A—C17A—C12A	121.39 (14)	N18B—C20B—H20F	109.5
C16A—C17A—C12A	119.51 (14)	H20D—C20B—H20F	109.5
C12A—N18A—C20A	115.13 (14)	H20E—C20B—H20F	109.5
C12A—N18A—C19A	113.73 (14)	N1—C21—H21A	109.5
C20A—N18A—C19A	109.71 (15)	N1—C21—H21B	109.5
N18A—C19A—H19A	109.5	H21A—C21—H21B	109.5
N18A—C19A—H19B	109.5	N1—C21—H21C	109.5
H19A—C19A—H19B	109.5	H21A—C21—H21C	109.5
N18A—C19A—H19C	109.5	H21B—C21—H21C	109.5
H19A—C19A—H19C	109.5	N1'—C21'—H21D	109.5
H19B—C19A—H19C	109.5	N1'—C21'—H21E	109.5
N18A—C20A—H20A	109.5	H21D—C21'—H21E	109.5
N18A—C20A—H20B	109.5	N1'—C21'—H21F	109.5
H20A—C20A—H20B	109.5	H21D—C21'—H21F	109.5
N18A—C20A—H20C	109.5	H21E—C21'—H21F	109.5
H20A—C20A—H20C	109.5	N1"—C21"—H21G	109.5
H20B—C20A—H20C	109.5	N1"—C21"—H21H	109.5
N1—C2B—C3B	112.5 (7)	H21G—C21"—H21H	109.5
N1—C2B—H2B1	109.1	N1"—C21"—H21I	109.5
C3B—C2B—H2B1	109.1	H21G—C21"—H21I	109.5
N1—C2B—H2B2	109.1	H21H—C21"—H21I	109.5
C3B—C2B—H2B2	109.1		
			
C2B—N1—C2A—C3A	179.6 (4)	C2A—N1—C2B—C3B	154.0 (5)
C21—N1—C2A—C3A	57.6 (4)	C21—N1—C2B—C3B	−82.8 (6)
N1—C2A—C3A—N4A	52.7 (4)	N1—C2B—C3B—N4B	−67.9 (12)
C2B'—N1'—C2A'—C3A'	−88.3 (7)	C2A'—N1'—C2B'—C3B'	178.0 (5)
C21'—N1'—C2A'—C3A'	148.9 (5)	C21'—N1'—C2B'—C3B'	−59.3 (8)
N1'—C2A'—C3A'—N4A	−56.3 (7)	N1'—C2B'—C3B'—N4B	−64.9 (7)
C2B"—N1"—C2A"—C3A"	172.9 (11)	C2A"—N1"—C2B"—C3B"	−169 (3)
C21"—N1"—C2A"—C3A"	−65.3 (12)	C21"—N1"—C2B"—C3B"	69 (3)
N1"—C2A"—C3A"—N4A	−57.4 (13)	N1"—C2B"—C3B"—N4B	32 (6)
C2A—C3A—N4A—S5A	129.7 (2)	C2B'—C3B'—N4B—S5B	173.4 (3)
C2A"—C3A"—N4A—S5A	178.4 (5)	C2B"—C3B"—N4B—S5B	142 (4)
C2A'—C3A'—N4A—S5A	−171.1 (3)	C2B—C3B—N4B—S5B	−175.0 (7)
C3A—N4A—S5A—O7A	175.3 (2)	C3B'—N4B—S5B—O6B	159.4 (4)
C3A"—N4A—S5A—O7A	162.2 (8)	C3B"—N4B—S5B—O6B	−175 (3)
C3A'—N4A—S5A—O7A	−161.3 (3)	C3B—N4B—S5B—O6B	−174.5 (8)
C3A—N4A—S5A—O6A	46.3 (2)	C3B'—N4B—S5B—O7B	−72.3 (4)
C3A"—N4A—S5A—O6A	33.2 (8)	C3B"—N4B—S5B—O7B	−46 (3)
C3A'—N4A—S5A—O6A	69.6 (3)	C3B—N4B—S5B—O7B	−46.2 (8)
C3A—N4A—S5A—C8A	−68.3 (2)	C3B'—N4B—S5B—C8B	42.0 (4)
C3A"—N4A—S5A—C8A	−81.4 (8)	C3B"—N4B—S5B—C8B	68 (3)
C3A'—N4A—S5A—C8A	−44.9 (3)	C3B—N4B—S5B—C8B	68.1 (8)
O7A—S5A—C8A—C9A	−130.22 (13)	O6B—S5B—C8B—C9B	139.95 (12)
O6A—S5A—C8A—C9A	−0.54 (16)	O7B—S5B—C8B—C9B	11.48 (14)
N4A—S5A—C8A—C9A	114.60 (14)	N4B—S5B—C8B—C9B	−106.28 (13)
O7A—S5A—C8A—C16A	50.70 (14)	O6B—S5B—C8B—C16B	−40.16 (15)
O6A—S5A—C8A—C16A	−179.61 (13)	O7B—S5B—C8B—C16B	−168.62 (12)
N4A—S5A—C8A—C16A	−64.48 (14)	N4B—S5B—C8B—C16B	73.62 (14)
C16A—C8A—C9A—C10A	0.2 (2)	C16B—C8B—C9B—C10B	−1.6 (2)
S5A—C8A—C9A—C10A	−178.88 (13)	S5B—C8B—C9B—C10B	178.28 (12)
C8A—C9A—C10A—C11A	−2.1 (3)	C8B—C9B—C10B—C11B	1.8 (2)
C9A—C10A—C11A—C17A	0.3 (3)	C9B—C10B—C11B—C17B	1.3 (2)
N18A—C12A—C13A—C14A	177.70 (15)	N18B—C12B—C13B—C14B	178.90 (14)
C17A—C12A—C13A—C14A	−5.0 (2)	C17B—C12B—C13B—C14B	1.6 (2)
C12A—C13A—C14A—C15A	−1.1 (2)	C12B—C13B—C14B—C15B	0.3 (2)
C13A—C14A—C15A—C16A	4.6 (2)	C13B—C14B—C15B—C16B	−0.4 (2)
C14A—C15A—C16A—C17A	−1.9 (2)	C14B—C15B—C16B—C8B	178.68 (14)
C14A—C15A—C16A—C8A	176.16 (15)	C14B—C15B—C16B—C17B	−1.4 (2)
C9A—C8A—C16A—C15A	−174.77 (15)	C9B—C8B—C16B—C15B	178.38 (15)
S5A—C8A—C16A—C15A	4.3 (2)	S5B—C8B—C16B—C15B	−1.5 (2)
C9A—C8A—C16A—C17A	3.3 (2)	C9B—C8B—C16B—C17B	−1.6 (2)
S5A—C8A—C16A—C17A	−177.66 (11)	S5B—C8B—C16B—C17B	178.54 (11)
C10A—C11A—C17A—C16A	3.3 (2)	C10B—C11B—C17B—C16B	−4.6 (2)
C10A—C11A—C17A—C12A	−179.40 (15)	C10B—C11B—C17B—C12B	176.78 (15)
C15A—C16A—C17A—C11A	173.25 (14)	C15B—C16B—C17B—C11B	−175.41 (14)
C8A—C16A—C17A—C11A	−4.9 (2)	C8B—C16B—C17B—C11B	4.5 (2)
C15A—C16A—C17A—C12A	−4.1 (2)	C15B—C16B—C17B—C12B	3.3 (2)
C8A—C16A—C17A—C12A	177.68 (14)	C8B—C16B—C17B—C12B	−176.76 (13)
C13A—C12A—C17A—C11A	−169.72 (15)	C13B—C12B—C17B—C11B	175.24 (14)
N18A—C12A—C17A—C11A	7.7 (2)	N18B—C12B—C17B—C11B	−2.1 (2)
C13A—C12A—C17A—C16A	7.6 (2)	C13B—C12B—C17B—C16B	−3.4 (2)
N18A—C12A—C17A—C16A	−175.00 (14)	N18B—C12B—C17B—C16B	179.21 (13)
C13A—C12A—N18A—C20A	16.3 (2)	C13B—C12B—N18B—C20B	−19.5 (2)
C17A—C12A—N18A—C20A	−161.01 (16)	C17B—C12B—N18B—C20B	157.82 (15)
C13A—C12A—N18A—C19A	−111.52 (18)	C13B—C12B—N18B—C19B	113.37 (17)
C17A—C12A—N18A—C19A	71.18 (19)	C17B—C12B—N18B—C19B	−69.35 (19)

### Hydrogen-bond geometry (Å, º)

**Table:**

*D*—H···*A*	*D*—H	H···*A*	*D*···*A*	*D*—H···*A*
N4*A*—H4*A*···O7*B*^i^	0.88 (2)	2.38 (2)	3.223 (2)	162 (2)
C20*A*—H20*B*···N4*A*^ii^	0.98	2.60	3.494 (2)	152
C2*B*"—H2*B*6···O6*B*^i^	0.99	2.54	3.359 (13)	140
C3*B*"—H3*B*5···O7*B*	0.99	2.37	2.93 (5)	115
N4*B*—H4*B*···O7*B*^i^	0.84 (2)	2.41 (2)	3.2108 (19)	159 (2)
C9*B*—H9*B*···O7*A*^i^	0.95	2.44	3.147 (2)	131
C21—H21*B*···O6*B*^i^	0.98	2.65	3.203 (4)	116

Symmetry codes: (i) −*x*+1, −*y*+1, −*z*+1; (ii) *x*, *y*+1, *z*.
