# Supplementary figures and images for: Crystal structure of 5,5′-bis­(di­methyl­amino)-N,N′-(3-methyl-3-aza­pentane-1,5-di­yl)di(naphthalene-1-sulfonamide)
Source: Acta Crystallogr E Crystallogr Commun. 2015 Nov 21;71(Pt 12):o959–60. doi: 10.1107/S2056989015021714 (PMC4719922; doi:10.1107/S2056989015021714)

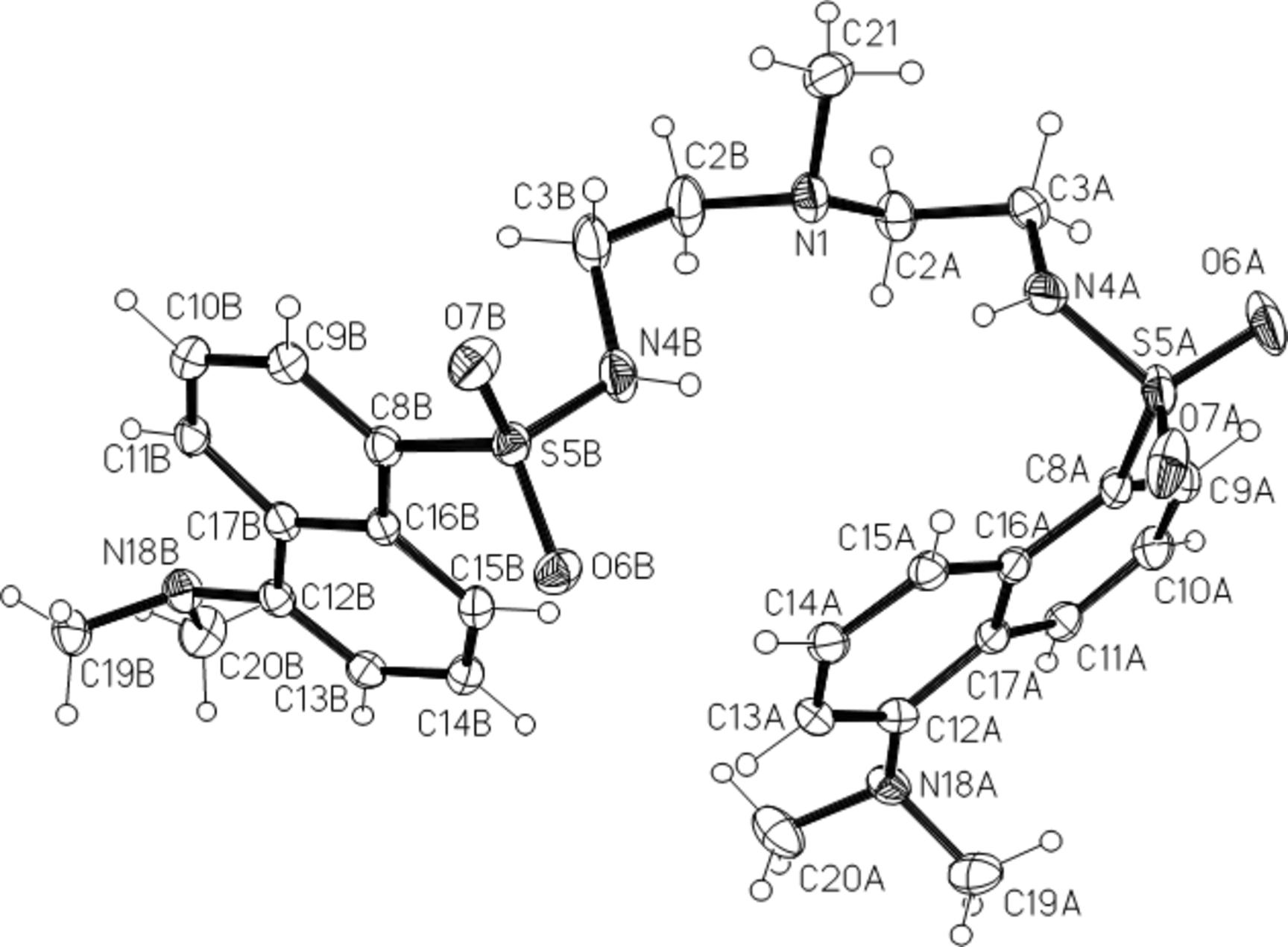

Supplement: Supplementary file 4 [file e-71-0o959-fig1.tif]

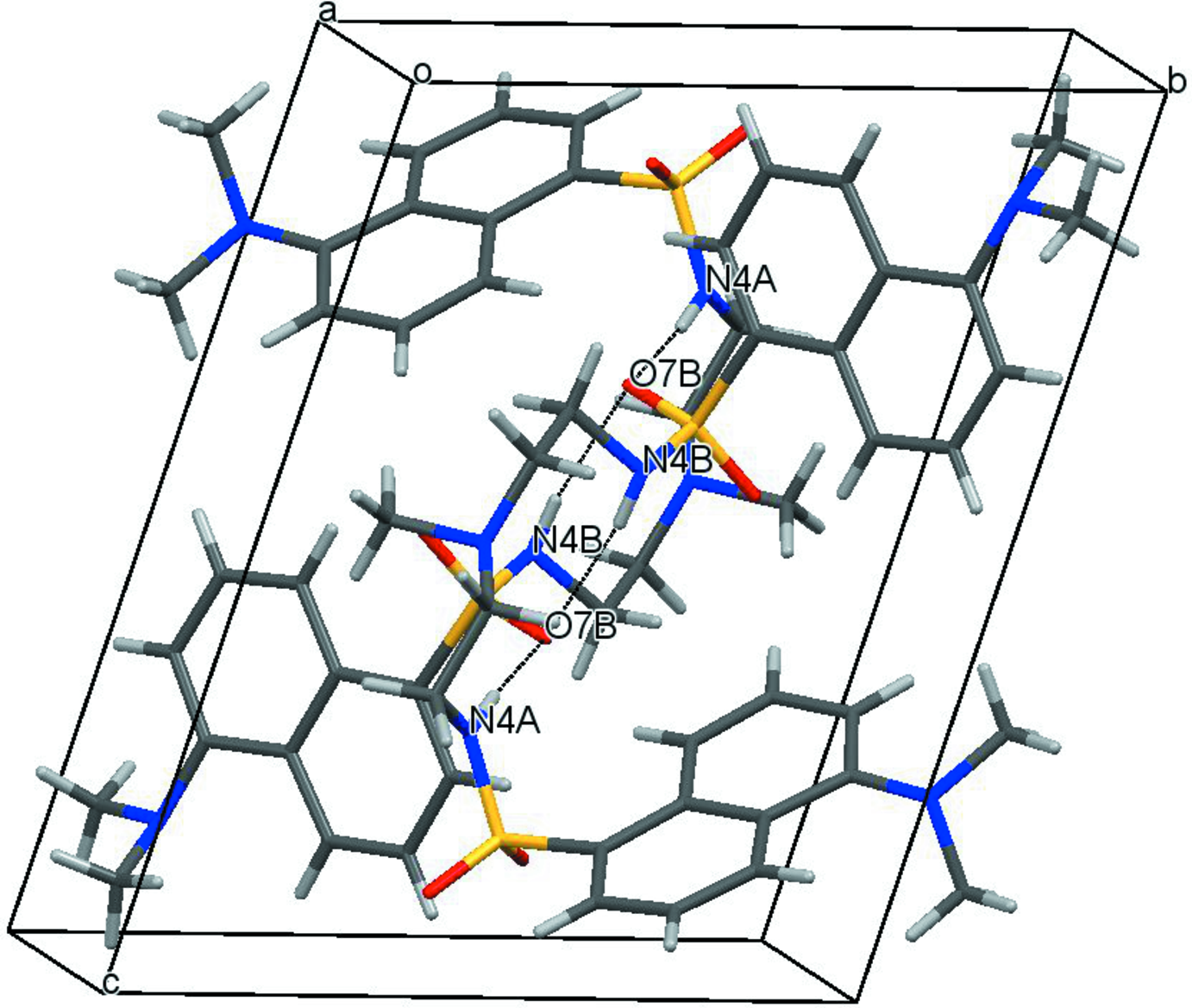

Supplement: Supplementary file 5 [file e-71-0o959-fig2.tif]
